# Supplementary material for: Photoacoustic imaging for non-invasive assessment of biomarkers of intestinal injury in experimental necrotizing enterocolitis
Source: Pediatr Res. 2024 Jun 24;97(1):169–77. doi: 10.1038/s41390-024-03358-2 (PMC11666804; doi:10.1038/s41390-024-03358-2)
Supplement: Supplementary file 5 — Supplementary materials 5 [file 41390_2024_3358_MOESM5_ESM.pdf]

Supplementary materials

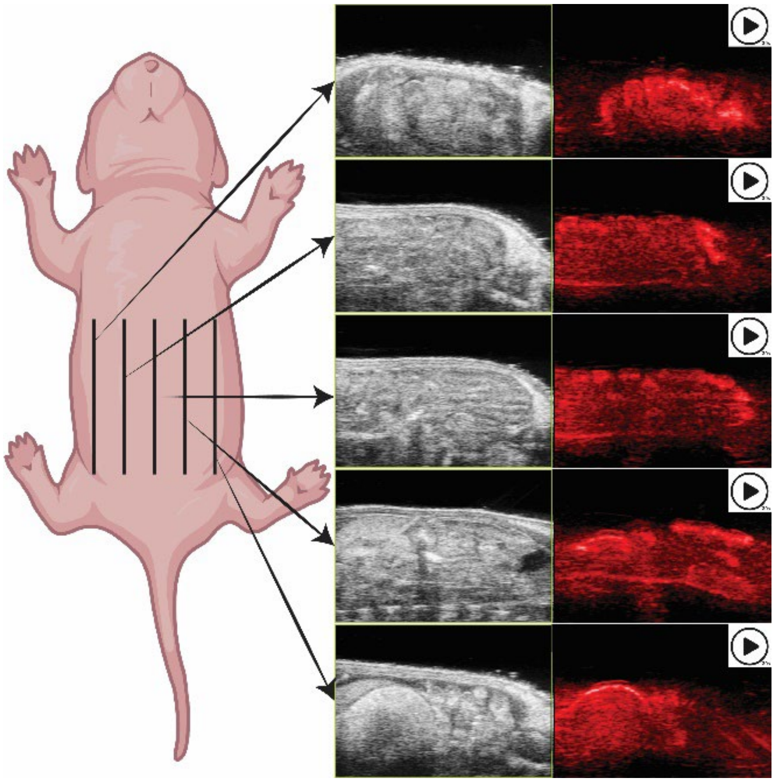

Supplemental Figure 1. Schematic of volumetric imaging data acquisition for intestinal motility analyses. Volumetric analysis of deformation was performed with 30 second cine US/PAI recordings at 5 equally spaced locations across the abdomen. Lines indicate locations of imaging planes throughout the intestinal volume.

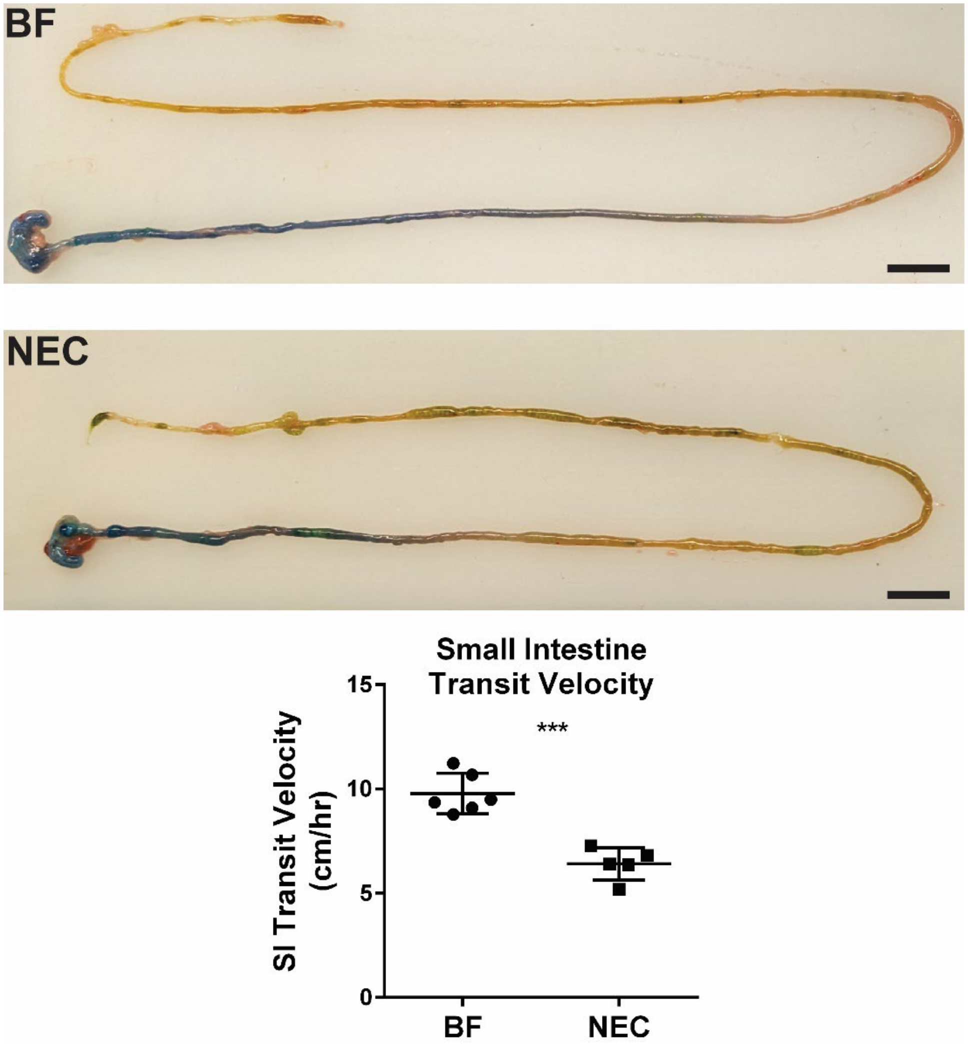

Supplemental Figure 2. (Top) Representative methylene blue images from small intestinal transit velocity assay. Scale bar = 1 cm. **(Bottom)** Transit assay showed a significant reduction in small intestinal transit velocity between BF and NEC. ($p<0.001$). Dye was found to transit significantly less distance in NEC pups as compared to healthy BF pups (6.41 cm ± 0.77 vs. 9.78 ± 0.97, $p < 0.001$) within the 1-hour transit time.

Supplemental Video 1. US and ICG-enhanced PAI imaging for intestinal motility in a representative 2-day old BF pup

Supplemental Video 2. US and ICG-enhanced PAI imaging for intestinal motility in a representative 2-day old NEC pup

Supplemental Video 3. US and ICG-enhanced PAI imaging for intestinal motility in a representative 4-day old BF pup

Supplemental Video 4. US and ICG-enhanced PAI imaging for intestinal motility in a representative 4-day old NEC pup
